# Neutrophil phenotypes in prolonged labor: Implications for therapeutic strategies

**DOI:** 10.1097/MD.0000000000040611

**Published:** 2024-11-15

**Authors:** Emmanuel Ifeanyi Obeagu, Getrude Uzoma Obeagu

**Affiliations:** a Department of Medical Laboratory Science, Kampala International University, Ishaka, Uganda; b School of Nursing Science, Kampala International University, Ishaka, Uganda.

**Keywords:** immune modulation, immunology of labor, inflammatory response, maternal-fetal interface, neutrophils, obstetric complications, prolonged labor

## Abstract

Prolonged labor, defined as labor extending beyond 20 hours for nulliparas and 14 hours for multiparas, poses significant risks to both maternal and neonatal health. The inflammatory response plays a crucial role in the pathophysiology of prolonged labor, with neutrophils being key players in this process. Neutrophils, the most abundant leukocytes, exhibit diverse phenotypes and functions in response to prolonged labor, influencing both the onset and progression of labor through their inflammatory actions. Classical neutrophils (N1) are involved in acute inflammatory responses, aiding in tissue remodeling and labor onset, but their prolonged activation can lead to tissue damage. Regulatory neutrophils (N2), which produce anti-inflammatory cytokines, help resolve inflammation and facilitate labor progression. Low-density granulocytes and aged neutrophils, associated with chronic inflammation and impaired function respectively, contribute to labor complications. The balance among these neutrophil phenotypes is crucial for maintaining a controlled inflammatory response during labor. Therapeutic strategies targeting neutrophil recruitment, NETosis, and cytokine production hold promise for managing prolonged labor. Modulating chemokine pathways, regulating NET formation, and balancing cytokine profiles may reduce inflammation and improve labor outcomes. Further research into the mechanisms of neutrophil regulation and the development of targeted therapies is essential for mitigating the adverse effects of prolonged labor and enhancing maternal and neonatal health.

## 1. Introduction

Prolonged labor, also known as labor dystocia, is a significant complication in obstetrics that poses substantial risks to both the mother and the fetus. Defined as labor that exceeds 20 hours in first-time mothers (nulliparas) and 14 hours in women who have previously given birth (multiparas), prolonged labor can result in a range of adverse outcomes, including increased rates of cesarean delivery, postpartum hemorrhage, infection, and neonatal distress. Understanding the underlying mechanisms that contribute to prolonged labor is crucial for developing effective therapeutic strategies to mitigate these risks.^[1]^ The onset and progression of labor are highly dependent on a complex interplay of hormonal, mechanical, and immunological factors. Among these, the inflammatory response plays a pivotal role. Inflammation is essential for the initiation of labor, facilitating cervical ripening, membrane rupture, and uterine contractions. Neutrophils, the most abundant leukocytes in the bloodstream, are key players in this inflammatory process. Their ability to rapidly respond to inflammatory signals makes them critical to the initiation and progression of labor.^[2]^ Neutrophils are traditionally viewed as a homogeneous population of cells primarily involved in acute inflammatory responses. However, recent research has revealed significant heterogeneity among neutrophil populations, with distinct phenotypes exhibiting different functional properties. This diversity allows neutrophils to adapt to various physiological and pathological conditions, including labor. In the context of prolonged labor, understanding the specific roles and regulation of different neutrophil phenotypes is essential for identifying potential therapeutic targets.^[3]^ Classical neutrophils, also known as N1 neutrophils, are characterized by their rapid response to inflammatory signals, their ability to phagocytose pathogens, and their release of reactive oxygen species (ROS) and proteolytic enzymes. These functions are crucial for the initial stages of inflammation and tissue remodeling, processes that are essential for the onset of labor. However, in the setting of prolonged labor, excessive activation and sustained presence of N1 neutrophils can lead to tissue damage and exacerbation of inflammation, contributing to labor complications.^[4]^

Regulatory neutrophils, or N2 neutrophils, play a counterbalancing role by producing anti-inflammatory cytokines such as IL-10 and TGF-β. These neutrophils help resolve inflammation and restore tissue homeostasis. In the context of labor, a balance between N1 and N2 neutrophils is crucial. An imbalance favoring N1 neutrophils can prolong and exacerbate inflammation, while an increase in N2 neutrophils may aid in resolving inflammation and facilitating the progression of labor.^[5]^ Low-density granulocytes (LDGs) are a subset of neutrophils found in the low-density fraction of peripheral blood mononuclear cells. These cells are associated with chronic inflammatory conditions and are characterized by an altered phenotype, including increased production of type I interferons and enhanced NETosis (neutrophil extracellular trap formation). The presence of LDGs in prolonged labor may indicate a chronic inflammatory state, which can contribute to labor complications and adverse outcomes.^[2]^ Aged neutrophils, which have spent a prolonged time circulating in the bloodstream, exhibit distinct phenotypic and functional characteristics compared to freshly released neutrophils. These include reduced chemotactic ability, altered receptor expression, and decreased functional capacity. In the context of prolonged labor, the accumulation of aged neutrophils may impair the resolution of inflammation, contributing to labor dystocia. Therapeutic strategies aimed at modulating the lifespan and function of neutrophils may offer potential benefits in managing prolonged labor.^[3]^ Several mechanisms regulate neutrophil function during labor, including chemotaxis, NETosis, and cytokine production. Chemotactic factors such as IL-8, CXCL1, and CXCL2 guide neutrophil migration to the site of inflammation, a critical step in the inflammatory response. Dysregulation of these signals in prolonged labor can lead to excessive neutrophil infiltration and sustained inflammation. NETosis, the formation of neutrophil extracellular traps, can also play a dual role in host defense and tissue damage. Balancing the beneficial and detrimental effects of NETs is crucial in prolonged labor. Additionally, the cytokine profile produced by neutrophils, including pro-inflammatory and anti-inflammatory cytokines, modulates the overall inflammatory response.^[4]^ Targeting neutrophil recruitment through chemokine receptor or adhesion molecule inhibitors, regulating NETosis with DNase or other agents, and modulating cytokine profiles with cytokine inhibitors or anti-inflammatory agents are potential strategies. These approaches aim to balance the inflammatory response, reduce tissue damage, and facilitate labor progression, ultimately improving maternal and neonatal outcomes.^[5]^

## 2. Aim

The aim of this review is to provide a comprehensive understanding of neutrophil dynamics in prolonged labor, explore the immunological mechanisms underlying this condition, and identify potential therapeutic strategies for effective management.

## 3. Rationale

Prolonged labor, defined as labor lasting beyond the expected time frame for delivery, is a prevalent complication in obstetrics with significant maternal and neonatal consequences. It is associated with increased risks of cesarean delivery, instrumental interventions, maternal exhaustion, and neonatal distress. Neutrophils, as key components of the innate immune system, play a crucial role in the inflammatory processes of labor. Their functions include pathogen clearance, tissue remodeling, and the regulation of the inflammatory response. However, in the context of prolonged labor, these processes can become dysregulated, leading to excessive inflammation and adverse outcomes. A detailed understanding of neutrophil behavior during prolonged labor can reveal insights into the mechanisms driving prolonged labor and identify potential targets for therapeutic intervention. Despite the recognition of neutrophils as central players in inflammation, there is a lack of comprehensive understanding regarding their specific roles and regulatory mechanisms in prolonged labor. Key aspects such as the detailed profiles of neutrophil activation, the formation of neutrophil extracellular traps (NETs), and the interactions between neutrophils and other immune cells during prolonged labor remain underexplored. Addressing these gaps can lead to novel insights and therapeutic approaches for managing prolonged labor. The identification of specific immunological signatures and mechanisms involved in neutrophil dynamics offers the potential for targeted therapeutic strategies. For instance, therapies that modulate neutrophil recruitment, activation, and apoptosis, or that alter cytokine profiles, could be developed to manage prolonged labor effectively. By understanding these mechanisms, researchers can design interventions that address the root causes of prolonged labor rather than just managing symptoms.

Current diagnostic approaches for prolonged labor often rely on clinical observations and mechanical interventions. Advanced biomarkers and diagnostic tools based on neutrophil dynamics could offer more precise methods for early diagnosis and risk assessment. Identifying biomarkers associated with neutrophil activation and inflammation could help in predicting the likelihood of prolonged labor and guiding appropriate management strategies. Prolonged labor can lead to a range of complications for both mother and child, including increased risk of infections, uterine atony, and adverse neonatal outcomes. Improved management of inflammation and labor progression can enhance maternal recovery and neonatal health, reducing the incidence of labor-related complications. Current clinical guidelines for managing prolonged labor are based on general practices rather than specific immunological insights. A better understanding of neutrophil dynamics can inform the development of evidence-based clinical guidelines and protocols tailored to the inflammatory aspects of prolonged labor. This can lead to more effective and individualized treatment approaches for patients experiencing prolonged labor. Exploring neutrophil dynamics in prolonged labor opens the door to novel therapeutic approaches that go beyond conventional treatments. For example, interventions targeting specific neutrophil functions or manipulating the inflammatory environment through novel drugs or biologics could be developed. This research could lead to innovative therapies that specifically address the underlying causes of prolonged labor. Studying neutrophil dynamics in prolonged labor fosters interdisciplinary research opportunities, integrating knowledge from immunology, obstetrics, endocrinology, and molecular biology. Collaborative efforts across these fields can lead to a more comprehensive understanding of prolonged labor and the development of innovative solutions.

## 4. Review methodology

### 4.1. Search strategy

#### 4.1.1. Literature search

A comprehensive literature search was performed using multiple electronic databases to ensure a thorough collection of relevant studies. The databases searched included:

- *PubMed*: For peer-reviewed biomedical literature.
- *Embase*: For comprehensive coverage of drug research and clinical guidelines.
- *Web of Science*: For a broad range of scientific disciplines.
- *Scopus*: For multidisciplinary research articles.
- *Cochrane Library*: For systematic reviews and clinical trials.

#### 4.1.2. Search terms

The search strategy involved the use of a combination of keywords and MeSH terms related to neutrophils and prolonged labor. The search terms included:

- Neutrophils
- Prolonged Labor
- Labor Duration
- Inflammation
- Neutrophil Extracellular Traps (NETs)
- Labor Complications
- Immune Response
- Pro-inflammatory Cytokines

#### 4.1.3. Search process

The search was conducted from [Start Date] to [End Date], and the search results were initially screened for relevance based on titles and abstracts. The full texts of potentially relevant articles were then reviewed for inclusion.

### 4.2. Selection criteria

#### 4.2.1. Inclusion criteria

Studies were included based on the following criteria:

- *Study Type*: Original research articles, systematic reviews, and clinical trials.
- *Population*: Studies involving human subjects or clinical data related to prolonged labor.
- *Intervention*: Research focusing on neutrophil dynamics, inflammatory responses, or therapeutic strategies related to prolonged labor.
- *Outcome Measures*: Studies that reported on neutrophil behavior, inflammation markers, or therapeutic outcomes in the context of prolonged labor.

#### 4.2.2. Exclusion criteria

Studies were excluded based on the following criteria:

- *Animal Studies*: Research not involving human subjects.
- *Non-English Publications*: Studies not published in English or without available English translations.
- *Irrelevant Topics*: Articles unrelated to neutrophils, prolonged labor, or labor management.

### 4.3. Neutrophil biology and functionality

Neutrophils, also known as polymorphonuclear leukocytes (PMNs), are the most abundant type of white blood cells in the human bloodstream, comprising 50% to 70% of the total leukocyte population. They are an essential component of the innate immune system, providing the first line of defense against infections by rapidly responding to inflammatory signals. Neutrophils are produced in the bone marrow and have a short lifespan, typically circulating in the blood for 6 to 8 hours before migrating to tissues where they survive for 1 to 2 days.^[6,7]^ Neutrophil development, or granulopoiesis, occurs in the bone marrow through a well-defined process involving several stages. The process begins with hematopoietic stem cells, which differentiate into myeloblasts. Myeloblasts then progress through the promyelocyte, myelocyte, metamyelocyte, and band cell stages before becoming mature neutrophils. This maturation process is tightly regulated by various growth factors and cytokines, including granulocyte colony-stimulating factor.^[8]^ Neutrophils are highly motile cells capable of rapidly migrating to sites of infection or injury in response to chemotactic signals. Chemokines such as interleukin-8 (IL-8), CXCL1, and CXCL2 are key mediators of neutrophil chemotaxis. Upon activation, neutrophils adhere to the vascular endothelium through interactions with adhesion molecules such as selectins and integrins. They then transmigrate across the endothelial barrier into the affected tissues, guided by a gradient of chemotactic factors.^[9,10]^ Once at the site of infection or injury, neutrophils become activated and exhibit enhanced phagocytic capabilities. They engulf and internalize pathogens and debris through a process known as phagocytosis. Neutrophils contain various granules, including primary (azurophilic), secondary (specific), and tertiary (gelatinase) granules, which are packed with antimicrobial proteins and enzymes. Upon phagocytosis, these granules fuse with the phagosome, releasing their contents to kill and digest the engulfed material.^[11]^

A critical aspect of neutrophil function is the production of ROS through the respiratory burst. This process is mediated by the enzyme complex NADPH oxidase, which generates superoxide anions that are subsequently converted into other ROS, such as hydrogen peroxide and hypochlorous acid. ROS play a vital role in the antimicrobial activity of neutrophils by directly killing pathogens and modulating the inflammatory response.^[12]^ In addition to phagocytosis and ROS production, neutrophils can form neutrophil extracellular traps (NETs), which are web-like structures composed of decondensed chromatin and antimicrobial proteins. NETs trap and kill pathogens extracellularly, preventing their spread. The formation of NETs, known as NETosis, can be induced by various stimuli, including microbial components, inflammatory cytokines, and activated platelets. While NETs play a protective role, excessive NET formation can contribute to tissue damage and exacerbate inflammation.^[13]^ Neutrophils are not only effector cells but also active participants in the modulation of the immune response. They produce a wide array of cytokines and chemokines that influence the activity of other immune cells. Pro-inflammatory cytokines such as tumor necrosis factor-alpha (TNF-α), interleukin-1 beta (IL-1β), and interleukin-6 (IL-6) amplify the inflammatory response, while anti-inflammatory cytokines such as interleukin-10 (IL-10) help resolve inflammation and restore tissue homeostasis.^[14]^ The resolution of inflammation involves the timely apoptosis (programmed cell death) and clearance of neutrophils. Apoptotic neutrophils are recognized and phagocytosed by macrophages in a process called efferocytosis. This clearance is essential for preventing the release of toxic neutrophil contents into the surrounding tissue, which can cause further damage and prolong inflammation. Dysregulation of neutrophil apoptosis and clearance can lead to chronic inflammation and tissue injury.^[15]^

Recent research has revealed that neutrophils are a heterogeneous population, exhibiting functional plasticity and distinct phenotypes depending on the context. Classical N1 neutrophils are pro-inflammatory and involved in pathogen clearance, while regulatory N2 neutrophils have anti-inflammatory properties and promote tissue repair. LDGs and aged neutrophils are other subsets with unique characteristics.^[16]^ The diverse functions and phenotypes of neutrophils present numerous opportunities for therapeutic intervention in inflammatory conditions, including prolonged labor. Targeting specific neutrophil functions, such as chemotaxis, NETosis, or cytokine production, can help modulate the inflammatory response. Therapies aimed at balancing pro-inflammatory and anti-inflammatory neutrophil activities may reduce tissue damage and improve outcomes in conditions characterized by dysregulated inflammation. Continued research into neutrophil biology and functionality is essential for developing effective therapies for inflammatory diseases and conditions.^[9,17]^

### 4.4. Neutrophil dynamics in prolonged labor

The dynamics of neutrophils during labor, particularly in cases of prolonged labor, are complex and multifaceted. Prolonged labor is often characterized by an extended and sometimes dysregulated inflammatory response. Neutrophils play a crucial role in the initiation and progression of labor through their involvement in inflammation, tissue remodeling, and immune response. Neutrophil recruitment to the uterine tissues is a critical step in the labor process. During labor, the uterine environment produces various chemotactic signals, including IL-8, CXCL1, and CXCL2, which guide neutrophils to the site of inflammation. In prolonged labor, there is often an overproduction or sustained presence of these chemokines, leading to excessive neutrophil infiltration. This prolonged recruitment can contribute to sustained inflammation, potentially exacerbating labor difficulties.^[18]^ Once recruited to the uterine tissues, neutrophils become activated and exhibit enhanced functional capabilities. In prolonged labor, the activation state of neutrophils is often heightened, resulting in increased production of ROS and the release of proteolytic enzymes from granules. While these actions are essential for tissue remodeling and pathogen clearance, their excessive activity can lead to tissue damage and exacerbate inflammatory responses, complicating the labor process.^[19]^ Neutrophil extracellular traps (NETs) are web-like structures composed of decondensed chromatin and antimicrobial proteins that can trap and kill pathogens extracellularly. During prolonged labor, the formation of NETs is often upregulated. While NETs play a protective role by preventing the spread of infection, their excessive formation can contribute to tissue damage and inflammation. The balance between beneficial and detrimental effects of NETs is crucial in managing prolonged labor.^[20]^ Neutrophils produce a wide array of cytokines that modulate the inflammatory response. In prolonged labor, there is often an imbalance in the cytokine profile, with an overproduction of pro-inflammatory cytokines such as TNF-α, IL-1β, and IL-6. This cytokine imbalance sustains the inflammatory environment, which can delay labor progression and contribute to complications. The anti-inflammatory cytokines, such as IL-10, are also crucial for resolving inflammation and facilitating labor progression. Therapeutic strategies aimed at restoring cytokine balance may help manage prolonged labor.^[21]^

Neutrophils interact with various other immune cells, including macrophages, dendritic cells, and lymphocytes, to coordinate the immune response during labor. These interactions are crucial for a balanced immune response. In prolonged labor, dysregulation of these interactions can lead to sustained inflammation and tissue damage. Understanding these cellular interactions can provide insights into potential therapeutic interventions to modulate the immune response and facilitate labor progression.^[22]^ Neutrophil heterogeneity plays a significant role in the dynamics of prolonged labor. Classical N1 neutrophils are pro-inflammatory and involved in pathogen clearance, while regulatory N2 neutrophils have anti-inflammatory properties and promote tissue repair. In prolonged labor, an imbalance favoring N1 neutrophils can sustain inflammation and contribute to labor complications. LDGs and aged neutrophils, with their unique characteristics, also play roles in the inflammatory environment of prolonged labor. Therapeutic strategies targeting specific neutrophil subsets may help restore balance and improve labor outcomes.^[23]^ Hormonal changes during labor significantly influence neutrophil dynamics. Hormones such as oxytocin, estrogen, and progesterone modulate neutrophil function and their interaction with other immune cells. In prolonged labor, hormonal imbalances can affect neutrophil recruitment, activation, and cytokine production, contributing to labor difficulties. The resolution of inflammation during labor involves the timely apoptosis and clearance of neutrophils. In prolonged labor, dysregulation of neutrophil apoptosis can lead to an accumulation of activated neutrophils, prolonging the inflammatory response and contributing to tissue damage. Efferocytosis, the process by which apoptotic neutrophils are cleared by macrophages, is crucial for resolving inflammation. Therapeutic strategies aimed at promoting neutrophil apoptosis and clearance may help manage prolonged labor.^[24]^ Modulating neutrophil recruitment through chemokine receptor or adhesion molecule inhibitors, regulating NET formation with agents such as DNase, and balancing cytokine profiles with cytokine inhibitors or anti-inflammatory agents are promising strategies. Additionally, targeting specific neutrophil subsets and promoting their apoptosis and clearance may help restore inflammatory balance and facilitate labor progression. Further research and clinical trials are needed to evaluate the efficacy and safety of these therapeutic interventions in managing prolonged labor.

### 4.5. Mechanisms of neutrophil regulation in prolonged labor

Neutrophils, as pivotal players in the innate immune response, exhibit a range of functions that are finely regulated to maintain a balance between effective host defense and tissue integrity. In the context of prolonged labor, understanding the mechanisms of neutrophil regulation is crucial for identifying potential therapeutic targets to mitigate complications. Neutrophil recruitment to the site of inflammation is primarily driven by chemotaxis, the directed movement of cells towards a chemical gradient of attractants. In labor, the uterus and cervix release various chemokines, including interleukin-8 (IL-8), CXCL1, and CXCL2, which bind to chemokine receptors on neutrophils such as CXCR1 and CXCR2. These interactions activate intracellular signaling pathways, leading to changes in the cytoskeleton and directed migration towards the source of chemokines. In prolonged labor, dysregulation of these chemotactic signals can result in excessive neutrophil infiltration, sustaining inflammation and contributing to labor complications.^[18]^ Upon reaching the site of inflammation, neutrophils are activated through interactions with pro-inflammatory cytokines (e.g., TNF-α, IL-1β), microbial products, and damage-associated molecular patterns (DAMPs). This activation triggers a series of intracellular signaling cascades, including the activation of NF-κB and MAPK pathways, which enhance the neutrophils’ antimicrobial functions. Activated neutrophils release ROS, proteolytic enzymes, and antimicrobial peptides. While these responses are essential for combating infections and facilitating tissue remodeling, their excessive activation in prolonged labor can lead to tissue damage and sustained inflammation.^[19]^ Neutrophil extracellular traps (NETs) are web-like structures composed of decondensed chromatin and antimicrobial proteins that neutrophils release to trap and kill pathogens extracellularly. The formation of NETs, known as NETosis, can be triggered by various stimuli, including microbial products, cytokines, and activated platelets. In prolonged labor, an overproduction of NETs can exacerbate inflammation and contribute to tissue damage. Regulating NET formation through agents such as DNase, which degrades extracellular DNA, may help mitigate these adverse effects.^[20]^ Neutrophils produce a variety of cytokines that influence the inflammatory response. Pro-inflammatory cytokines like TNF-α, IL-1β, and IL-6 amplify inflammation, while anti-inflammatory cytokines such as IL-10 help resolve inflammation and promote tissue healing. In prolonged labor, an imbalance in cytokine production, with a predominance of pro-inflammatory cytokines, can sustain the inflammatory response and delay labor progression. Therapeutic strategies aimed at modulating cytokine production, such as cytokine inhibitors or agents that enhance anti-inflammatory cytokine production, may help restore balance and facilitate labor progression.^[21]^

Neutrophils interact with various other immune cells, including macrophages, dendritic cells, and lymphocytes, to coordinate the immune response. These interactions are mediated through cell surface receptors, cytokines, and chemokines. For example, neutrophils can influence macrophage activation and function through the release of cytokines and direct cell-to-cell contact. In prolonged labor, dysregulation of these interactions can lead to sustained inflammation and impaired resolution of the inflammatory response. Understanding these cellular interactions can provide insights into potential therapeutic interventions to modulate the immune response and facilitate labor progression.^[22]^ The resolution of inflammation involves the timely apoptosis and clearance of neutrophils. Apoptotic neutrophils express “eat me” signals such as phosphatidylserine on their surface, which are recognized by macrophages. The process of engulfing and digesting apoptotic cells by macrophages is called efferocytosis. In prolonged labor, impaired neutrophil apoptosis and clearance can lead to the accumulation of activated neutrophils, prolonging inflammation and contributing to tissue damage. Therapeutic strategies aimed at promoting neutrophil apoptosis and enhancing efferocytosis may help manage prolonged labor.^[23]^ Hormonal changes during labor significantly influence neutrophil dynamics. Hormones such as oxytocin, estrogen, and progesterone modulate neutrophil function and their interaction with other immune cells. For instance, oxytocin has been shown to have anti-inflammatory effects and can influence neutrophil chemotaxis and activation. In prolonged labor, hormonal imbalances can affect neutrophil recruitment, activation, and cytokine production, contributing to labor difficulties. Neutrophil heterogeneity includes various subsets with distinct functional properties. Classical N1 neutrophils are pro-inflammatory and involved in pathogen clearance, while regulatory N2 neutrophils have anti-inflammatory properties and promote tissue repair. LDGs and aged neutrophils, with their unique characteristics, also play roles in the inflammatory environment of prolonged labor. Therapeutic strategies targeting specific neutrophil subsets may help restore balance and improve labor outcomes.^[24]^

### 4.6. Immunological signatures in prolonged labor

Prolonged labor, a significant complication in obstetrics, is characterized by extended inflammatory responses that can lead to adverse outcomes for both mother and child. Immunological signatures, defined as distinct patterns of immune cell behavior, cytokine production, and molecular markers, offer valuable insights into the underlying mechanisms of prolonged labor. Identifying these signatures can help in understanding the pathophysiology of prolonged labor and developing targeted therapeutic interventions. In prolonged labor, neutrophil activation and infiltration are markedly elevated. This is evidenced by increased levels of chemokines such as IL-8, CXCL1, and CXCL2 in the uterine tissues and cervical mucus. These chemokines attract neutrophils to the site of inflammation, resulting in their accumulation and sustained activation. Elevated levels of neutrophil-derived enzymes, such as myeloperoxidase (MPO) and elastase, are common immunological signatures in prolonged labor, reflecting heightened neutrophil activity and degranulation.^[25,26]^ The cytokine milieu in prolonged labor is characterized by a predominance of pro-inflammatory cytokines. Elevated levels of TNF-α, IL-1β, IL-6, and IL-8 are typically observed, indicating a robust inflammatory response. These cytokines are produced by both neutrophils and other immune cells present in the uterine environment. The imbalance between pro-inflammatory and anti-inflammatory cytokines, with reduced levels of IL-10, highlights the difficulty in resolving inflammation, contributing to the prolongation of labor.^[27]^ Increased formation of neutrophil extracellular traps (NETs) is a distinct immunological signature in prolonged labor. NETs, which are composed of decondensed chromatin and antimicrobial proteins, trap and kill pathogens but can also exacerbate inflammation. Elevated levels of circulating cell-free DNA, a marker of NETosis, and histone-DNA complexes are often found in the blood and amniotic fluid of women experiencing prolonged labor. This indicates excessive NET formation and a potential target for therapeutic intervention.^[28]^ Interactions between neutrophils and other immune cells, such as macrophages and lymphocytes, are altered in prolonged labor. Neutrophils can influence macrophage polarization, promoting a pro-inflammatory M1 phenotype over the anti-inflammatory M2 phenotype. This shift contributes to sustained inflammation and tissue damage. Additionally, interactions with T cells, particularly the increased presence of Th17 cells, which produce IL-17, further amplify the inflammatory response. These altered immune cell interactions are key immunological signatures in prolonged labor.^[29]^

Hormonal changes during labor significantly impact immune responses. In prolonged labor, hormonal imbalances, such as reduced levels of progesterone and altered oxytocin signaling, influence neutrophil activity and cytokine production. Progesterone typically exerts an anti-inflammatory effect, promoting immune tolerance and reducing neutrophil activation. Its reduction in prolonged labor removes this inhibitory effect, contributing to heightened inflammation. Monitoring hormonal levels and their interaction with immune responses provides critical insights into the immunological signatures of prolonged labor.^[30]^ The presence and activity of specific neutrophil subsets, including classical N1 and regulatory N2 neutrophils, as well as LDGs, are crucial in shaping the immunological landscape of prolonged labor. An imbalance favoring pro-inflammatory N1 neutrophils and LDGs, which are associated with chronic inflammation, is often observed. These subsets produce higher levels of ROS and pro-inflammatory cytokines, sustaining the inflammatory environment. Identifying these subsets and their activity patterns can help in understanding the immunological signatures of prolonged labor.^[31]^ Various molecular markers are indicative of prolonged labor and its associated immunological signatures. Elevated levels of acute phase proteins, such as C-reactive protein (CRP) and serum amyloid A (SAA), reflect systemic inflammation. Additionally, increased expression of adhesion molecules, such as ICAM-1 and VCAM-1, on endothelial cells facilitates neutrophil migration and retention in the uterine tissues. Monitoring these molecular markers can provide valuable diagnostic and prognostic information.^[32]^ Transcriptomic analyses reveal distinct gene expression profiles associated with prolonged labor. Upregulation of genes involved in inflammatory pathways, such as NF-κB, MAPK, and JAK-STAT signaling, is commonly observed. Genes encoding pro-inflammatory cytokines, chemokines, and adhesion molecules are also overexpressed. Conversely, genes associated with anti-inflammatory responses and tissue repair are downregulated. These gene expression profiles constitute important immunological signatures that can guide therapeutic development.^[33]^ Targeting key elements of the inflammatory response, such as inhibiting specific cytokines (e.g., IL-1β, TNF-α) or modulating neutrophil activity and NET formation, can help in managing prolonged labor. Hormonal therapies to restore balance and the use of anti-inflammatory agents to counteract excessive inflammation are promising strategies. Further research into these immunological signatures will enhance our ability to develop targeted and effective treatments for prolonged labor.^[23]^

### 4.7. Neutrophil phenotypes as therapeutic targets

Neutrophils exhibit diverse phenotypic profiles and functionalities, making them potential targets for therapeutic intervention in conditions such as prolonged labor.^[34]^ Exploring these phenotypes opens avenues for novel therapeutic strategies. Interventions aimed at modulating the activation states of neutrophils could be beneficial. Regulating the balance between pro-inflammatory and anti-inflammatory phenotypes or promoting resolution phenotypes might mitigate excessive inflammation associated with prolonged labor.^[35,36]^ Strategies targeting the recruitment of neutrophils to the maternal-fetal interface could potentially alter the inflammatory milieu.^[37]^ This might involve inhibiting specific chemotactic signals or adhesion molecules crucial for neutrophil migration to the site of inflammation. Boosting the functional capacities of neutrophils, such as enhancing their phagocytic activity or promoting the release of anti-inflammatory mediators, could aid in resolving inflammation and facilitating labor progression in cases of prolonged labor.^[38]^

Utilizing neutrophils as vectors for delivering therapeutic agents or modulating immune responses represents an innovative approach. Engineering neutrophils to carry anti-inflammatory payloads or immunomodulatory molecules might enable targeted immunomodulation at the maternal-fetal interface.^[39]^ As research identifies distinct neutrophil subsets associated with prolonged labor, developing therapies tailored to target specific neutrophil phenotypes or subsets may offer more precise and effective interventions.^[40]^ Immunotherapeutic approaches aimed at reprogramming or regulating the immune response involving neutrophils could potentially restore immune homeostasis and resolve inflammation in cases of prolonged labor.^[41]^ Combining therapies that target different aspects of neutrophil behavior, such as recruitment, activation, and functionality, might yield synergistic effects and enhance therapeutic efficacy in managing prolonged labor.^[42]^ Striving for personalized therapeutic strategies based on the specific immunological signatures and neutrophil phenotypes observed in individual cases of prolonged labor could optimize treatment outcomes.^[43]^ Harnessing the potential of neutrophil phenotypes as therapeutic targets necessitates a deeper understanding of their diverse roles and behaviors in the context of prolonged labor.^[44]^ Developing interventions that precisely modulate neutrophil functions and immune responses may offer promising avenues for mitigating the inflammatory cascade associated with prolonged labor and improving obstetric outcomes.

### 4.8. Clinical implications

Clinicians can utilize biomarkers such as elevated levels of IL-8, TNF-α, and neutrophil-derived enzymes like myeloperoxidase (MPO) and elastase to identify patients at risk for prolonged labor. Monitoring these biomarkers in real-time can provide insights into the inflammatory status and progression of labor, allowing for timely interventions. The heterogeneity of neutrophil responses and their regulatory mechanisms in prolonged labor highlight the need for personalized therapeutic strategies. By identifying specific neutrophil subsets and their activity patterns, clinicians can tailor treatments to target the dominant pro-inflammatory pathways. For instance, patients exhibiting excessive neutrophil extracellular trap (NET) formation may benefit from therapies that inhibit NETosis, such as DNase treatment.^[45]^ The imbalance between pro-inflammatory and anti-inflammatory cytokines in prolonged labor suggests a potential role for anti-inflammatory therapies. Drugs that inhibit pro-inflammatory cytokines like TNF-α, IL-1β, and IL-6 could help reduce excessive inflammation. Additionally, promoting the production of anti-inflammatory cytokines such as IL-10 might aid in resolving inflammation and facilitating labor progression. The use of corticosteroids, which have broad anti-inflammatory effects, could also be considered, although their impact on labor and fetal outcomes needs careful evaluation.^[46]^ Hormonal regulation plays a critical role in neutrophil dynamics during labor. Hormonal therapies aimed at restoring balance, such as progesterone supplementation, may help modulate neutrophil activity and cytokine production. Oxytocin, commonly used to induce labor, also has immunomodulatory effects that could be harnessed to manage prolonged labor. Understanding the interplay between hormones and neutrophil function can guide the use of hormonal treatments to optimize labor outcomes.^[47]^

Modulating neutrophil recruitment and activation offers another therapeutic avenue. Inhibitors of chemokine receptors (e.g., CXCR1, CXCR2) and adhesion molecules (e.g., ICAM-1, VCAM-1) can reduce excessive neutrophil infiltration and activation in the uterine tissues. Such therapies could help limit tissue damage and sustain inflammation, promoting a more favorable environment for labor progression.^[48]^ Promoting neutrophil apoptosis and enhancing their clearance (efferocytosis) by macrophages can help resolve inflammation in prolonged labor. Therapies that enhance the signaling pathways for apoptosis, such as those involving the Fas receptor or Bcl-2 family proteins, could be beneficial. Additionally, agents that stimulate macrophage activity and efferocytosis, like macrophage colony-stimulating factor (M-CSF), might aid in the timely clearance of neutrophils, reducing prolonged inflammation.^[49]^ Strategies to minimize tissue damage caused by excessive neutrophil activity are crucial in managing prolonged labor. Antioxidants that neutralize ROS produced by activated neutrophils can help protect tissues. Protease inhibitors that target neutrophil-derived enzymes like elastase and matrix metalloproteinases (MMPs) can reduce tissue degradation and support the structural integrity of the uterine environment.^[48]^ Effective management of neutrophil dynamics and inflammatory responses in prolonged labor has direct implications for maternal and neonatal outcomes. Reducing excessive inflammation and tissue damage can lower the risk of complications such as infections, uterine atony, and cesarean deliveries. For neonates, a controlled inflammatory environment reduces the likelihood of preterm birth-related complications, improving overall health and development.^[47]^ Incorporating knowledge of neutrophil dynamics into clinical guidelines and protocols can enhance the management of prolonged labor. Standardized protocols for monitoring inflammatory biomarkers, assessing neutrophil activity, and applying targeted therapies can improve clinical outcomes. Training healthcare providers on the importance of immunological factors in labor progression and how to manage them effectively is essential for optimizing care.^[49]^ Table 1 shows Molecular Mechanisms of Neutrophil Dynamics in Prolonged Labor (provided by the authors).

**Table 1:** Molecular mechanisms of neutrophil dynamics in prolonged labor

Molecular mechanism	Description	Role in prolonged labor
Neutrophil Recruitment	Chemotactic signals such as IL-8 and CXCL8 guide neutrophils to the labor site	Increased recruitment can exacerbate inflammation and delay labor progression
Neutrophil Activation	Activation markers include CD11b and CD66b, which reflect neutrophil readiness	Activated neutrophils release inflammatory mediators, contributing to prolonged labor
NET Formation	Neutrophil extracellular traps (NETs) trap pathogens but can also cause tissue damage	Excessive NET formation leads to chronic inflammation and prolonged labor
Cytokine Production	Key cytokines include IL-1β, TNF-α, and IL-6 that modulate inflammatory responses	Elevated levels of these cytokines promote and sustain inflammation in prolonged labor
Neutrophil Apoptosis	Apoptosis is regulated by pro-apoptotic and anti-apoptotic signals like Fas/FasL	Dysregulation of apoptosis contributes to prolonged inflammation and labor complications
Protease Release	Proteases such as MPO and elastase are released during neutrophil degranulation	Proteases contribute to tissue remodeling and inflammation, which can prolong labor
Oxidative Stress	ROS generation during neutrophil activation can damage tissues	Increased oxidative stress leads to tissue damage and exacerbates prolonged labor
Chemokine Receptors	Receptors such as CXCR1 and CXCR2 mediate neutrophil migration and activation	Dysregulation of these receptors can lead to excessive neutrophil infiltration and inflammation
Inflammatory Feedback Loops	Feedback mechanisms regulate cytokine production and neutrophil responses	Disrupted feedback loops can lead to sustained inflammation and exacerbate prolonged labor
Neutrophil-Immune Cell Interactions	Interactions with macrophages and T-cells modulate the inflammatory response	Aberrant interactions can prolong inflammation and impact labor progression

ROS = reactive oxygen species.

### 4.9. Future directions

One of the foremost directions for future research is the discovery and validation of advanced biomarkers for prolonged labor. Utilizing high-throughput technologies such as proteomics, genomics, and metabolomics can uncover novel biomarkers that offer greater specificity and sensitivity. These biomarkers could help in early detection, monitoring, and prognosis of prolonged labor, allowing for timely and targeted interventions. Further investigation into the roles and regulatory mechanisms of different neutrophil subsets, including N1 and N2 neutrophils, LDGs, and aged neutrophils, is essential. Research should focus on the signaling pathways, gene expression profiles, and epigenetic modifications that govern neutrophil subset differentiation and activity.^[50]^ Neutrophil extracellular traps (NETs) play a dual role in host defense and tissue damage. Future research should delve into the specific triggers and regulatory pathways of NETosis in prolonged labor. Investigating how NET formation is modulated by hormonal changes, microbial infections, and immune cell interactions can provide insights into developing targeted therapies to inhibit excessive NET formation while preserving their protective functions.^[51]^ Developing and testing immunomodulatory therapies that can precisely modulate the inflammatory response in prolonged labor is a critical area of future research. This includes cytokine inhibitors, anti-inflammatory agents, and therapies that promote neutrophil apoptosis and clearance. Preclinical and clinical trials are needed to evaluate the efficacy and safety of these therapies, considering their potential impacts on both maternal and fetal health. The interplay between hormonal regulation and neutrophil dynamics during labor warrants further exploration. Research should focus on how specific hormones such as oxytocin, estrogen, and progesterone influence neutrophil function and the inflammatory environment. Hormonal interventions that can restore balance and promote a favorable labor progression need to be rigorously tested in clinical settings. Advancing personalized medicine approaches in the management of prolonged labor involves integrating individual patient data, including genetic, epigenetic, and immune profiling. Future research should aim to develop predictive models and decision-support tools that can personalize treatment plans based on a patient’s unique immunological and hormonal profile. This approach can optimize therapeutic efficacy and minimize adverse outcomes.^[52–62]^

Longitudinal studies that track immune responses and labor progression over time can provide valuable insights into the dynamics of prolonged labor. Leveraging big data and machine learning algorithms to analyze large datasets from diverse populations can identify patterns and predictors of prolonged labor. This can enhance our understanding of the condition and guide the development of targeted interventions. Future research should also focus on the implications of maternal neutrophil dynamics and inflammatory responses on neonatal health. Studies exploring how prolonged labor and the associated inflammatory environment affect neonatal outcomes, such as immune development, infection susceptibility, and long-term health, are crucial. This can inform interventions that not only address maternal health but also optimize neonatal well-being.^[54]^ Investigating alternative therapies, such as phytochemicals, probiotics, and dietary supplements, that have immunomodulatory properties could offer additional avenues for managing prolonged labor. Research should focus on understanding the mechanisms through which these therapies influence neutrophil function and inflammation and evaluating their potential benefits and risks in clinical trials. Promoting interdisciplinary research and collaboration between obstetricians, immunologists, endocrinologists, and researchers from other relevant fields can accelerate advancements in understanding and managing prolonged labor. Collaborative efforts can lead to a more holistic approach, integrating various perspectives and expertise to develop comprehensive strategies for addressing this complex condition.^[54]^

## 5. Conclusion

Prolonged labor is characterized by sustained inflammation, driven in part by the overactivation and dysregulation of neutrophils. These immune cells, through mechanisms such as chemotaxis, activation, NET formation, and cytokine production, significantly influence the labor process and its outcomes. Identifying the immunological signatures specific to prolonged labor has not only enhanced our understanding of its pathophysiology but also highlighted potential therapeutic targets. The clinical implications of these findings are vast, offering avenues for early diagnosis, personalized treatment approaches, and improved maternal and neonatal outcomes. Targeted therapies that modulate neutrophil activity, hormonal treatments that restore balance, and interventions that enhance the resolution of inflammation hold promise for managing prolonged labor more effectively. Incorporating these insights into clinical practice can lead to more precise and effective interventions, ultimately improving the health and well-being of both mothers and their babies.

## Author contributions

**Conceptualization:** Emmanuel Ifeanyi Obeagu.

**Methodology:** Emmanuel Ifeanyi Obeagu, Getrude Uzoma Obeagu.

**Supervision:** Emmanuel Ifeanyi Obeagu.

**Validation:** Getrude Uzoma Obeagu.

**Visualization:** Emmanuel Ifeanyi Obeagu, Getrude Uzoma Obeagu.

**Writing – original draft:** Emmanuel Ifeanyi Obeagu, Getrude Uzoma Obeagu.

**Writing – review & editing:** Emmanuel Ifeanyi Obeagu, Getrude Uzoma Obeagu.
